# Drought Stress Influences the Growth and Physiological Characteristics of *Solanum rostratum* Dunal Seedlings From Different Geographical Populations in China

**DOI:** 10.3389/fpls.2021.733268

**Published:** 2021-11-16

**Authors:** Hailun Yu, Xueyong Zhao, Wenda Huang, Jin Zhan, Yuanzheng He

**Affiliations:** ^1^Naiman Desertification Research Station, Northwest Institute of Eco-Environment and Resources, Chinese Academy of Sciences (CAS), Lanzhou, China; ^2^Urat Desert-Grassland Research Station, Northwest Institute of Eco-Environment and Resources, Chinese Academy of Sciences (CAS), Lanzhou, China; ^3^University of Chinese Academy of Sciences, Beijing, China

**Keywords:** *Solanum rostratum* Dunal, invasive species, drought stress, functional traits, lipid peroxidation, antioxidant enzyme activity

## Abstract

Extensive studies have shown that the success of invasive plants in large environmental gradients can be partly attributed to related factors, including phenotypic plasticity and rapid evolution. To enhance their ability to compete and invade, invasive plants often show higher morphological and physiological plasticity to adapt to different habitat conditions. In the past two decades, invasive species have expanded to some new habitats in North and Northwest China, including arid oasis agricultural zones, which are disturbed by human activities, and the ecosystem itself is very fragile. To evaluate the ecological adaptability of invasive plants widely distributed in North and Northwest China, we studied the physiological response and tolerance mechanism of different geographical populations of *Solanum rostratum* Dunal to different drought-stress gradients in extremely arid regions (Xinjiang population) and semi-arid regions (Inner Mongolia population). The results showed that with the aggravation of drought stress, *S. rostratum* from different geographical populations adopted different physiological mechanisms to drought stress. Xinjiang population was mostly affected by root/shoot ratio and chlorophyll fluorescence characteristics, showing higher plasticity in the net and total photosynthetic rates, while the Inner Mongolia population mainly relied on the accumulation of osmotic adjustment substances, higher leaf dry matter content, and increased malondialdehyde to cope with drought stress. Based on these results, we concluded that the physiological responses of *S. rostratum* invading different habitats in northern China to drought stress were significantly different. The drought resistance of the Xinjiang population was higher than that of the Inner Mongolia population. In general, *S. rostratum* can be widely adapted to both harsh and mild habitats through phenotypic plasticity, threatening agricultural production and ecological environment security in northern China.

## Introduction

Biological invasion is increasing globally and causing significant impacts on ecosystem functioning. Single invasive species can alter ecosystem processes and adversely affect the environment. Invasive plant species have been proven to displace native species, change vegetation structure, reduce native biodiversity (Pyšek and Richardson, 2008; Hejda et al., 2009), undermine functioning of the whole ecosystem, and cause significant economic losses (Zavaleta, 2000; Powell et al., 2013). Previous studies indicated that exotic plants can speedily respond to environmental selection pressure (Losos et al., 1997; Bone and Farres, 2001); however, there is significant adaptive genetic differentiation among different populations of invasive plants on a short time scale (Lee, 2002; Maron et al., 2004). For instance, studies on the invasive plant *Brumus tectorum* showed that the populations growing under different water conditions had adaptive evolution to their habitats, making their local fitness significantly higher than other populations (Rice and Mack, 1991). The invasive plant *Impatiens glandulifera*, which is distributed in distinct latitudes, showed differing genetic traits among growth characteristics, such as plant height, basal diameter, biomass, and phenological characteristics (flowering time) (Kollmann and Bañuelos, 2004). Local adaptations of invasive organisms have attracted the attention of ecologists (Maron et al., 2004). Local adaptations are an important factor to promote the invasive species to adapt to the new environment, settle, and spread successfully (Sakai et al., 2001; Bossdorf et al., 2005).

*Solanum rostratum* Dunal is native to Mexico and the southwestern United States (Whalen, 1979), which was listed in the first batch of “National key management alien invasive species list” in China in 2013. *S. rostra*tum has a strong breeding and competitive ability and forms a dominant population and encroached ecosystems in an invasive manner (Gao et al., 2005), usually grows aggressively in habitats disturbed (Vallejo Marín et al., 2013), so as it is treated as a noxious weed (Bassett and Munro, 1986). Since being discovered in Liaoning province in the 1980s, it has spread to many provinces in northern China (Zhao et al., 2013) and disturbed habitats, including sandy land, abandoned fields, oasis farmland edge, both sides of highways, roadsides, and riversides (Chen et al., 2013). These distribution areas are thousands of kilometers away, and the water conditions vary greatly. How *S rostratum* can survive, spread, and form communities and compete with native species in such vastly different habitats is an important research direction in the mechanism of its regulation and physiological response to drought stress.

To enhance their ability to compete and invade, alien plants often show higher morphological and physiological plasticity to adapt to different habitat conditions (Peng and Xiang, 1999). Plants adopt diverse and complex physiological and biochemical adaptive mechanisms to deal with drought stress (Neumann, 2008). In all these mechanisms, antioxidant enzyme system is an important stress resistance mechanism for plants to cope with various environmental challenges, plays a vital role in the process of plant adaptation (Prasad, 1997; Hernández et al., 2001; Bor et al., 2003) and alleviation of damage caused by drought stress (Wu et al., 2014, 2015). Environmental stress can promote the increase of antioxidant enzyme activity to a certain extent (Wu et al., 2011). Invasive plant Flaveria bidentis resists the stress of low and medium concentration cadmium by increasing the activities of superoxide dismutase (SOD), peroxidase (POD) and catalase (CAT), and shows a strong tolerance to Cd stress (Zhang et al., 2020). Invasive plant *Eupatorium odoratum* is based on the continuous increase of protective enzyme activities, such as superoxide dismutase (SOD), dehydroalbumin reductase (DHAR) and APX, as well as a higher ratio of root to shoot and water use efficiency to resist high and low temperature stress and drought stress (Lu et al., 2006; Wu et al., 2007). Soil moisture is an important limiting factor affecting plant growth and distribution (Matesanz et al., 2015); therefore, it is of great significance to study the drought tolerance of invasive plants to predict their distribution range and prevention.

Due to environmental factors have a great impact on the invasion process and mechanism, the same invasive plant may show different invasion mechanisms through phenotypic plasticity and adaptive evolution in different environments (Wang et al., 2011). Invasive plant *Abutilon theophrasti* originally from Asia invaded in North America. In soybean field, the invasion is realized through increased plasticity of stem length, but the decreased plasticity in corn field, through adaptation mechanism (Weining, 2000). Plants occupying a wide ecological range and diverse habitats often have phenotypic variations in morphology, phenology, physiology, and life history, according to changes in local ecological environment factors (Blossey and Notzold, 1995; Siemann and Rogers, 2001; Ebeling et al., 2008). However, it is unclear if this variation is a simple response of plants to climatic conditions, or an adaptive evolution based on local environmental conditions. This needs further experimental proof, and a homogeneous nursery experiment is one of the most widely used methods (Moloney et al., 2009).

## Materials and Methods

### Sampling Area

**FIGURE 1:**
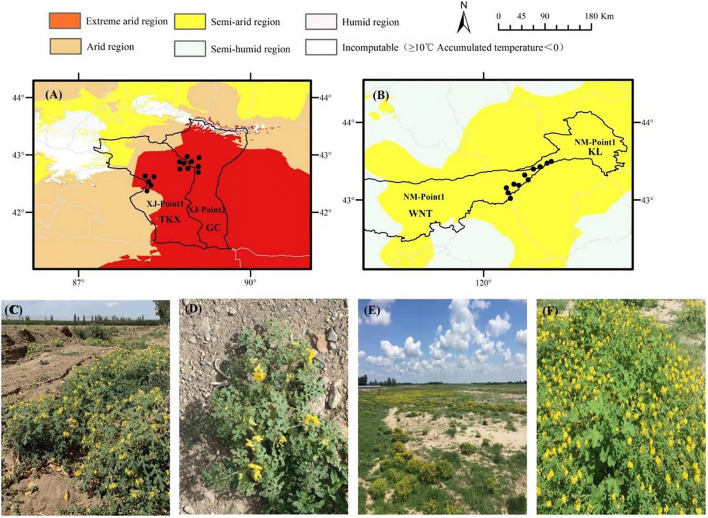
Locations of seed collection sites for *Solanum rostratum* used in this study. The collection sites of Xinjiang were located in an extremely arid climate region **(A)**, mainly distributed in Tuokexun County (**A**, XJ-Point1, abbreviated as TKX) and Gaochang district (**A**, XJ-Point2, abbreviated as GC) of Turpan city. The distribution habitat included farmland fields **(C)** and roadside communities **(D)**. The collection sites in Inner Mongolia were in a semi-arid climate region **(B)**, mainly distributed in Ongniud Banner (**B**, NM-Point1, abbreviated as WNT) and Kailu County (**B**, NM-Point2, abbreviated as KL). The distribution habitat included river courses in the dry season **(E)** and grassland communities **(F)**.

From June to September 2018, four naturally growing populations of *S. rostratum* were collected from Xinjiang and Inner Mongolia. The field capacity of the Xinjiang population was 21.1% in Tuokexun County (TKX) and 19.2% in Gaochang district (GC), measured on August 7; the field capacity of Inner Mongolia population was 17.81% in Kailu County (KL) and 14.33% in Ongniud Banner (WNT), measured on August 20. The geographical location, habitat and related messages of the groups are shown in Figure 1 and Table 1.

**TABLE 1:** Origin of materials and number of samples for different populations of *Solanum rostratum* in China.

Population	Location(country)	Number of plants	Latitude(°N)	Longitude(°E)	Habitats	Voucher
TKX1	Tuokexun	15	42°32′24″	88°13′18″	farmland	Yu20190101-(1-15)
TKX2	Tuokexun	15	42°38′16″	88°8′52″	roadside	Yu20190102-(1-15)
TKX3	Tuokexun	10	42°36′57″	88°19′26″	farmland	Yu20190103-(1-10)
TKX4	Tuokexun	10	42°45′51″	88°53′52″	roadside	Yu20190104-(1-10)
TKX5	Tuokexun	8	42°52′44″	88°44′42″	bareland	Yu20190105-(1-8)
GC1	Gaochang	15	42°57′25″	89°13′22″	roadside	Yu20190201-(1-15)
GC2	Gaochang	13	42°57′39″	88°53′34″	roadside	Yu20190202-(1-13)
GC3	Gaochang	15	42°53′24″	89°01′16″	bareland	Yu20190203-(1-15)
GC4	Gaochang	15	42°42′25″	89°6′21″	bareland	Yu20190204-(1-15)
GC5	Gaochang	15	42°41′34″	89°10′11″	roadside	Yu20190205-(1-15)
WNT1	Ongniud	15	43°22′28″	119°31′22″	river course in dry season	Yu20190301-(1-15)
WNT2	Ongniud	15	43°9′11″	120°18′10″	roadside	Yu20190302-(1-15)
WNT3	Ongniud	15	43°5′16″	120°19′37″	roadside	Yu20190303-(1-15)
WNT4	Ongniud	15	43°11′34″	120°25′26″	river course in dry season	Yu20190304-(1-15)
WNT5	Wengniute	15	43°19′51″	120°32′16″	river course in dry season	Yu20190305-(1-15)
KL1	Kailu	13	43°24′08″	120°39′3″	grassland	Yu20190401-(1-13)
KL2	Kailu	15	43°25′55″	120°44′24″	grassland	Yu20190402-(1-15)
KL3	Kailu	15	43°29′22″	120°49′30″	farmland	Yu20190403-(1-15)
KL4	Kailu	9	43°32′27″	120°53′34″	roadside	Yu20190404-(1-9)

The Tuokexun population was collected from Tuokexun County, Turpan Region, which belongs to the extremely arid continental climate, in warm variable zones with low rainfall and high evaporation. The climate is hot in summer; the annual average precipitation is only 9.8 mm, and this area gets the least precipitation in China. This region is windy, with an average of 84 gale days. The collection sites are located on farmland fields, and *S. rostratum* is an important dominant species in the population. The relative abundance of *S. rostratum* was more than 83%, accompanied by *Setaria viridis* (L.) Beauv, *Tribulus terrestris* (L.), *Digitaria sanguinalis* (L.) Scop., *Alhagi sparsifolia* Shap., and the invasive plant *Xanthium italicum* Moretti.

The Gaochang population was collected from Gaochang district, Turpan Region, which also belongs to the extremely arid continental climate zone. The extreme high temperature in summer is 49.6°C, and the surface temperature is more than 70°C. This place is often called “fire land” with low precipitation and high evaporation, and a large daily and annual range of temperature differences. The collection sites are located on both sides of the road, so the soil particle size is larger than Tuokexun sites. *S. rostratum* is a single dominant species in this population; the relative abundance of *S. rostratum* was more than 90%, accompanied by *Tribulus terrestris* (L.), *Halogeton glomeratus*, *Setaria viridis* (L.) Beauv, *Alhagi sparsifolia* Shap., and *Hibiscus trionum* L.

The Ongniud population was collected in Ongniud Banner, which is located on the west edge of the Horqin sandy land, belonging to semiarid climate and the upper reaches of the West Liao River basin. Most of the areas belong to semi-arid and semi-humid climate zones. The annual precipitation is more than 370 mm, and summer precipitation accounts for 75% of the total annual precipitation. The collection sites are located on river course in dry season, and the *S. rostratum* is an important dominant species in this population; the relative abundance of *S. rostratum* was more than 70%, accompanied by *Agriophyllum squarrosum* (L.) Mo, *Amaranthus retroflexus* L., *Chloris virgata* Sw., *Echinochloa crusgalli* (L.) Beauv, and *Digitaria sanguinalis* (L.) Scop.

The Kailu population was collected in Kailu County, located in the temperate continental semiarid monsoon climate zone belonging to the lower reaches of the West Liao River basin. The annual precipitation is more than 338.3 mm. The collection sites are located on the grassland of the Laoha River (tributary of Liaohe River), and the soil is moist. *S. rostratum* grows alongside *Chloris virgata* Sw., *Leymus chinensis* (Trin.) Tzvel., *Artemisia halodendron* Turcz. et Bess., *Digitaria sanguinalis* (L.) Scop, *Tribulus terrestris* (L.), *Agriophyllum squarrosum* (L.) Moq., and *Echinochloa crusgalli* (L.) Beauv, forming a community with high diversity. The relative abundance of *S. rostratum* is 57%.

### Experimental Design and Treatments

A homogeneous nursery was formed in the greenhouse of the Plant Physiology Laboratory of Agricultural and Ecological Department, Northwest Institute of Eco-Environment and Resources (NIEER), Chinese Academy of Sciences, from June to August 2019. The seeds, which had been treated by low temperature (4°C) for 48 h, were seeded into the cultivation grid containing mixed soil (volume ratio, garden soil: sand = 1:1). Fifteen seeds were planted in each grid at a depth of 0.5–1.0 cm. After germination to the emergence of the first true leaf, each population were selected and transplanted into plastic pots (15 cm in diameter and 18 cm in depth); among them, Xinjiang population (TKX and GC sites) planted 60 seedlings respectively, and Inner Mongolia population (WNT and KL sites) planted 70 seedlings respectively. We planted 260 plants in 130 pots, before the breeding period, these plants were deeply planted in the soil of the biological garden.

Seedlings were subjected to different water conditions using polyethylene glycol (PEG 6000). Four water treatments were designed for the drought stress gradient, the mass concentration of PEG 6000 was 0, 150, 200 and 300 g/L respectively, corresponding solution osmotic potential (Ψo) was about 0 MPa (control, CK), − 0.38 MPa (light drought, LD), − 0.61 MPa (moderate drought, MD) and −1.20 MPa (severe drought, SD) (Michel and Kaufmann, 1973). When the third true leaf of the seedlings was unfolded, seedlings with the same growth trend were selected for further analyses.

When the third true leaf of seedlings of each geographical population expands, seedlings with the same growth trend are selected. Four treatments were set for each population, five replicates were set for each treatment, and three seedlings were selected for each replicate for drought stress treatment.

In drought treatment, plants were watered twice a week with PEG 6000 solution. All experiments were conducted in a controlled environment chamber under the following conditions: long-day photoperiod (14 h of light), 23°C during the day, 15°C at night, and 40–80% relative humidity. The experiments started on August 10 and ended on August 25 and were kept for 15 days.

### Determination of Plant Functional Traits

Plant height was measured with a steel tape and vernier caliper (the accuracy is 0.02 mm). Leaves that were free of diseases and fully extended were harvested and taken immediately to the laboratory. The fresh weight of the leaves was obtained on an electronic balance scale to the nearest one-millionth. The leaves were immersed in distilled water for 24 h. Excess water on the leaf surface was absorbed by filter paper, and the saturated fresh weight of the leaves was measured. The soaked leaves were then placed into an oven at 65°C and dried to a constant weight. These procedures were repeated three times. Leaf relative water content (LRWC) and leaf dry matter content (LDMC) (Izanloo et al., 2008) were also determined. The whole plant was divided into aboveground and underground parts, washed with distilled water, sterilized in 105°C ovens for 15 min, and dried at 65°C to a constant weight. The dry matter content of aboveground and underground parts was weighed to calculate the root/shoot ratio (R/S). The calculation formula of each value are shown in Supplementary Table 2.

### Determination of Chlorophyll Content and Chlorophyll Fluorescence Parameters

Mature spreading leaves at the top of the plants were cut and crushed. Extract solution (50 mL; *V*_ethanol_: *V*_acetone_ = 1:1) was added to 0.2 g of leaves. Extraction was performed at 25°C in the dark for 24 h, and absorbance was measured at 440, 645, and 663 nm to determine the content of chlorophyll in the leaves (Pavlovič, 2011).

Chlorophyll fluorescence parameters were determined by a chlorophyll fluorescence instrument (Hansatech, England) (Baker, 2008). The initial fluorescence (*F*_o_) was determined by irradiating the measuring light < 0.5 μmol/(m^2^⋅s), and the maximum fluorescence (*F*_m_) was detected by irradiation with saturated pulses [2800 μmol/(m^2^⋅s)] after dark adaptation for 20 min. Then open endogenous photochemical light for 5 min to determine the steady state fluorescence (*F*_s_). The saturated pulse [2800 μmol/(m^2^⋅s)] interval was set to 20 s to measure the maximum fluorescence (*F*_m_′) under light adaptation. *F*_v_ was calculated by subtracting *F*_o_ from *F*_m_. The calculation formula of the maximum photochemical efficiency and the photochemical quenching coefficient are shown in Supplementary Table 2.

### Determination of Malondialdehyde and Antioxidant Enzyme Activity

The content of malondialdehyde (MDA) was determined by the thiobarbituric acid (TBA) method (Wang et al., 2010). A 0.5-g leaf sample was added to 5 mL 10% trichloroacetic acid (TCA), and the homogenate was centrifuged for 10 min; 2 mL of supernatant was mixed with 2 mL of 0.6% TBA. After 10 min in a water bath at 100°C, the supernatant was cooled and centrifuged. The absorbance of the supernatant was measured at 450, 532, and 600 nm. The calculation formula are shown in Supplementary Table 2.

The extraction method of Mishra et al. (2006) was used for antioxidant enzyme activity determination. Briefly, a 0.5-g leaf sample was placed in a precooled mortar (ice bath), and 1-mL phosphate buffer was added to grind the sample into a slurry. Buffer (5 mL) was added, and the solution was centrifuged at 4°C for 10 min. The supernatant was the crude enzyme solution, which was used to measure the activity of antioxidant enzymes. The activity of superoxide dismutase (SOD) was measured spectrophotometrically at 560 nm by the nitro blue tetrazole (NBT) method (Stewart and Bewley, 1980); the 50% inhibition of NBT photoreduction was regarded as an enzyme activity unit. The activity of POD was measured by the guaiacol method, and the absorbance of crude enzyme solution at 470 nm was determined by absorption spectrophotometry (Shah and Nahakpam, 2012). The activity of catalase (CAT) was detected by the absorbance of the crude enzyme at 240 nm. One CAT unit was the amount of A_240_ that decomposed 1 μmol H_2_O_2_ per min at 25°C (Aebi, 1984).

### Determination of Osmotic Regulation Substances

The content of free proline was measured by acid ninhydrin colorimetry. The absorbance of the supernatant was measured spectrophotometrically at 520 nm (Bates et al., 1973). The content of soluble protein (SP) was determined by the Coomassie Brilliant Blue G-250 staining method and measured with a spectrophotometer at 595 nm (Bradford, 1976). The content of soluble sugars (SS) was determined by anthrone colorimetry and measured with a spectrophotometer at 620 nm (Buysse and Merckx, 1993). Proline was expressed as μg/g of fresh weight of the leaf (FW), and the soluble sugars and soluble proteins were expressed as mg/g of FW.

### Data Analysis

The collected sites and climatic regionalization method of four geographic populations of *S. rostratum* were established on the “China Meteorological background data set” provided by the Resource and Environment Science and Data Center using ArcGIS 10.2 in the GCS_WGS_1984 coordinate system.

A two-way analysis of variance was used to calculate the effect of drought stress on the functional and physiological indices of *S. rostratum*. Before variance analysis, the Shapiro Wilk test was used to test the validity of the normality hypothesis, as well as the Levene test to determine the homogeneity of variance. If variance analysis was satisfied, the effects of different populations and drought stress on the main and interactive effects of physiological indices of *S. rostratum* were tested by two factor ANOVA, and then the Duncan test was used to compare the effects. Principal component analysis (PCA) was used to analyze the physiological response indices of *S. rostratum* under different drought-stress conditions. SPSS 22.0 was used for data analysis; column diagrams were drawn with Origin software (Origin Pro 2021). The corresponding relationship and principal component analyses were carried out by R language software (version r3.6.3).

## Results

### Response of Plant Functional Traits to Drought Stress

**FIGURE 2:**
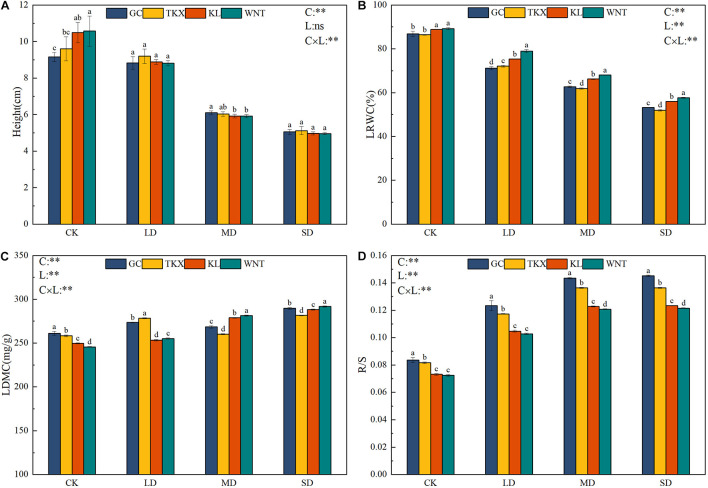
Change in functional traits of different geographical populations of *Solanum rostratum* under drought stress. Plant height [Height (cm)] **(A)**, leaf relative water content [LRWC (%)] **(B)**, leaf dry matter content [LDMC (mg/g)] **(C)**, and root shoot ratio [R/S (%)] **(D)** of treatments with the indicated polyethylene glycol concentrations. The blue column represents the GC site, yellow column represents the TKX site, red column represents to KL site, and green column represents the WNT site. Different lowercase letters indicate a significant difference under drought stress in *S. rostratum* populations, according to Duncan’s test (α = 0.05). C refers to the differences among drought-stress concentrations; L refers to the differences among each geographical population; C × L refers to the interaction between populations and concentration. The significant change in functional traits between the four collection sites was shown by the significance of population–stress treatment interactions. Asterisks indicate a significant difference (*, *p* ≤ 0.05; **, *p* ≤ 0.01; ***, *p* ≤ 0.001). Refer to statistics in Supplementary Table 1.

There was a significant interactive effect between different populations and drought-stress conditions on the functional traits of *S. rostratum*. Under the no stress condition, the height of the Inner Mongolia populations (KL: 10.5 cm; WNT: 10.7 cm) were significantly higher than that of the Xinjiang populations (TKX: 9.6 cm; GC: 9.2 cm) (*p* < 0.01). Compared with the control, in the MD condition, the height of WNT (5.92 cm) decreased 44.67%, KL (5.91 cm) decreased 43.71%, TKX (6.03 cm) decreased 37.19%, and GC (6.11 cm) decreased 33.58%. In the SD condition, the height of Inner Mongolia populations decreased (KL: 4.98 cm, decreased 52.57%; WNT: 4.96 cm, decreased 53.64%) more than that of the Xinjiang populations (TKX: 5.12 cm, decreased 46.47%; GC 5.05 cm, decreased 45.11%) (Figure 2A).

Leaf relative water content decreased with the increased drought-stress concentration (Figure 2B). Under no stress conditions, the LRWC of *S. rostratum* from the four sampling sites exceeded 80%, with the WNT population being nearly 90%. In the LD condition, the LRWC of the KL and WNT populations declined, but still exceeded 75%, which was significantly higher than the GC and TKX populations (*p* < 0.01). The four populations under stress showed a significant decrease in the overall LRWC, and Inner Mongolia populations decreased more than those from Xinjiang in the whole process of stress.

The response of LDMC to drought stress was significantly diverse (*p* < 0.01). Under CK conditions, the LDMC content of GC (261.1 mg/g) and TKX (258.3 mg/g) populations were higher than KL (249.7 mg/g) and WNT (245.6 mg/g). The LDMC of the four collection sites differed remarkably under LD conditions. The populations of Xinjiang (TKX: 278.4 mg/g; GC: 273.5 mg/g) had a significantly higher LDMC than the sites of Inner Mongolia (KL: 253.3 mg/g; WNT: 255.2 mg/g) (*p* < 0.01); however, under MD conditions, the LDMC of KL (278.9 mg/g) and WNT (281.5 mg/g) populations was largely increased, but GC (268.4 mg/g) and TKX (260.2 mg/g) decreased. The LDMC of the four collection sites increased under SD conditions, the WNT population had the highest and the TKX population had the lowest LDMC, and there was a significant difference among the four sites (*p* < 0.01) (Figure 2C).

With the increase of drought stress, the R/S of the four collection sites of *S. rostratum* showed an increasing trend (Figure 2D). Under CK, the R/S of Inner Mongolia populations (KL: 0.073; WNT: 0.073) was lower than that of Xinjiang populations (TKX: 0.082; GC: 0.084). Compared with the control, in LD stress, the R/S of the GC (0.139) population increased by more than 47.5%, which was significantly higher than that of the TKX (increased 43.67%), KL (increased 43.01%), and WNT populations (increased 41.78%). Under MD and SD stress, the R/S of the TKX (0.137) site increased significantly and was higher than other sites. The WNT (0.121) population had the lowest R/S under those stress conditions. The four populations under stress showed a significant increase under stress; the R/S of Xinjiang populations was always higher than that of Inner Mongolia populations under stress.

### Response of Chlorophyll Fluorescence to Drought Stress

**FIGURE 3:**
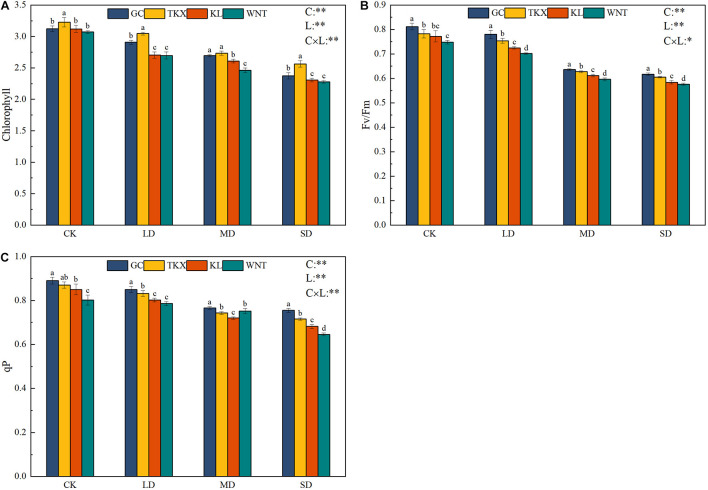
Effects of drought stress on the photosynthetic characteristics of different geographical populations of *Solanum rostratum*. Changes in leaf chlorophyll **(A)**, maximal chemical efficiency (*F*_v/_*F*_m_) **(B)**, and dynamics of photochemical quenching (*q*P) **(C)** of *S. rostratum* under different drought-stress concentrations. The blue column represents the GC site, yellow column represents the TKX site, red column represents to KL site, and green column represents the WNT site. Different lowercase letters indicate a significant difference under drought stress in *S. rostratum* populations, according to Duncan’s test (α = 0.05). C refers to the differences among drought-stress concentrations; L refers to the differences among each geographical population; C × L refers to the interaction between populations and concentration. The significant change in functional traits between the four collection sites was shown by the significance of population–stress treatment interactions. Asterisks indicate a significant difference (*, *p* ≤ 0.05; **, *p* ≤ 0.01; ***, *p* ≤ 0.001). Refer to statistics in Supplementary Table 1.

The chlorophyll content of the TKX site was higher than that in other sites under control conditions (*p* < 0.01). In LD stress, the KL and WNT populations showed a significant downward trend, and the chlorophyll content of the Xinjiang populations was higher than those of the Inner Mongolia populations. Under MD stress, the GC and TKX populations decreased significantly and were still higher than Inner Mongolia populations. Under SD stress, chlorophyll content of the TKX population was the highest, and compared with CK condition, chlorophyll content in Inner Mongolia populations (KL: decreased 26.09%; WNT: decreased 25.85%) decreased more than that in Xinjiang populations (GC: decreased 24.01%; TKX decreased 20.52%; *p* < 0.01; Figure 3A).

The maximum photochemical efficiency of *S. rostratum* seedings declined with increased drought stress and decreased significantly under MD drought stress, reaching the lowest efficiency under SD stress. Compared with the CK condition, the *F*_v_*/F*_m_ values of each *S. rostratum* population decreased to the lowest values under SD stress; these values decreased by 24.35% in KL, 24.01% in GC, 22.99% in WNT, and 22.63% in TKX (*p* < 0.01). Two-way ANOVA analysis showed that the interaction between populations and concentration was not significant (*p* < 0.05) (Figure 3B).

Compared with those under CK condition, the *q*P of the KL population under LD stress decreased (5.65%), and WNT decreased slightly (1.75%). Under MD stress, compared with the control, the *q*P of KL declined more than at other sites (*p* < 0.01). Under SD stress, the *q*P of each collection site decreased. Inner Mongolia populations decreased (KL decreased 19.76%, WNT decreased 19.45%) more than Xinjiang populations (GC decreased 15.17%, TKX decreased 17.75%). The decline rates were as follows: 27.6% in KL; 35.6% in WNT; 30.1% in TKX; and 31.1% in GC (Figure 3C).

### Oxidative Stress Responses and Antioxidant Enzyme Activity in Different Treatments

**FIGURE 4:**
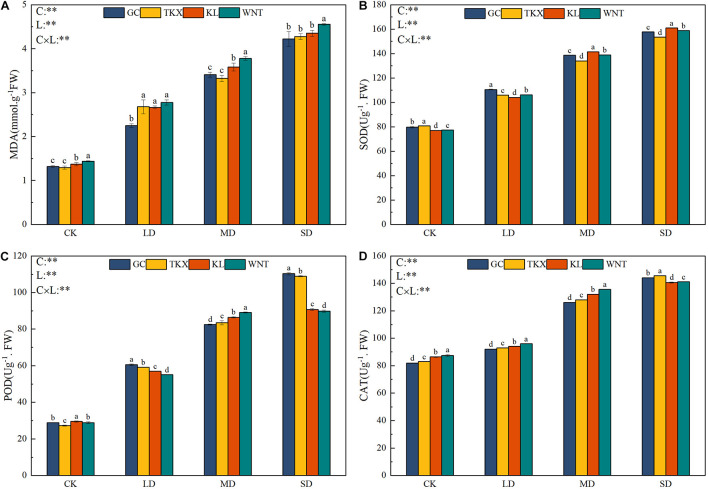
Effects of drought stress on malondialdehyde (MDA) accumulation and antioxidant enzyme activity of different geographical populations of *Solanum rostratum*. Changes in MDA **(A)**, superoxide dismutase (SOD) **(B)**, peroxidase (POD) **(C)**, and catalase (CAT) **(D)** of *S. rostratum* under different drought-stress concentrations. The blue column represents the GC site, yellow column represents the TKX site, red column represents to KL site, and green column represents the WNT site. Different lowercase letters indicate a significant difference under drought stress in *S. rostratum* populations, according to Duncan’s test (α = 0.05). C refers to the differences among drought-stress concentrations; L refers to the differences among each geographical population; C × L refers to the interaction between populations and concentration. The significant change in functional traits between the four collection sites was shown by the significance of population–stress treatment interactions. Asterisks indicate a significant difference (*, *p* ≤ 0.05; **, *p* ≤ 0.01; ***, *p* ≤ 0.001). Refer to statistics in Supplementary Table 1.

Under different concentrations of PEG, the content of MDA was positively correlated with PEG concentration, and the content of MDA increased with the increase of PEG concentration (Figure 4A). Under LD stress, the MDA content in the four populations of *S. rostratum* seedlings was more notable than that of the control. The increase in MDA content was highest in the TKX population (51.68%), which was not significantly different from that of the Inner Mongolia populations; however, the growth rate of the GC population was the lowest (41.39%). The MDA content of the Inner Mongolia populations was found to be remarkably higher than that of the Xinjiang populations, and differences with the corresponding control were observed under MD stress. Severe drought stress induced a remarkable increase in MDA content in each population compared with that in the control conditions, whereas the WNT population had a higher content than other sites (*p* < 0.05).

With the increase of drought stress, the SOD activity of *S. rostratum* seedlings showed an upward trend (Figure 4B). The SOD content of the Xinjiang populations was higher than that of the Inner Mongolia populations in control conditions. SOD activity of the GC population was increased higher (27.82%) than other sites in LD stress. The SOD activity of each site increased significantly (*p* < 0.001) compared with the control, while Inner Mongolia populations had higher activity than Xinjiang populations in MD and SD stress conditions.

The POD activity of *S. rostratum* increased along with increased drought stress (Figure 4C). The POD activity of the Inner Mongolia populations increased more than that of the Xinjiang populations under MD stress conditions. Under SD stress, POD of the Xinjiang populations (GC: 110.306 Ug^–1^. FW; TKX: 108.892 Ug^–1^. FW) increased significantly more than the Inner Mongolia populations (KL: 90.672 Ug^–1^. FW; WNT: 89.766 Ug^–1^. FW) (*p* < 0.001). Xinjiang sites mainly depended on increasing POD activity to resist the oxidative damage caused by drought stress.

The CAT activity of *S. rostratum* at each collection site increased significantly along with the increase in drought stress (*p* < 0.001). Under LD and MD stress, CAT activity of the Inner Mongolia populations was higher than that of the Xinjiang populations, and when the stress intensity was severe, CAT activity at the Xinjiang site was higher than that of the Inner Mongolia sites. Increased CAT activity was an adaptive strategy of the Xinjiang populations to resist the oxidative damage caused by severe drought stress (Figure 4D).

### Changes in Osmoregulatory Substances to Drought Intensity

**FIGURE 5:**
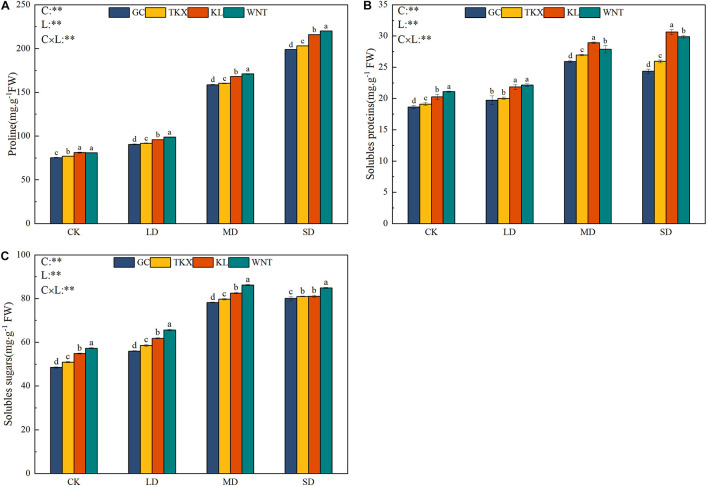
Effects of drought stress on the osmoregulatory substances of different geographical populations *Solanum rostratum*. Changes in proline **(A)**, soluble protein **(B),** and soluble sugar content **(C)** of *S. rostratum* under different drought-stress concentrations. The blue column represents the GC site, yellow column represents the TKX site, red column represents to KL site, and green column represents the WNT site. Different lowercase letters indicate a significant difference under drought stress in *S. rostratum* populations, according to Duncan’s test (α = 0.05). C refers to the differences among drought-stress concentrations; L refers to the differences among each geographical population; C × L refers to the interaction between populations and concentration. The significant change in functional traits between the four collection sites was shown by the significance of population–stress treatment interactions. Asterisks indicate a significant difference (*, *p* ≤ 0.05; **, *p* ≤ 0.01; ***, *p* ≤ 0.001). Refer to statistics in Supplementary Table 1.

With the increase of drought intensity, the content of proline in the stems and leaves of different geographical populations of *S. rostratum* increased gradually (Figure 5A). The free proline content in stems and leaves of *S. rostratum* in Inner Mongolia collection sites was higher than that in Xinjiang sites during the whole experimental period. The free proline content in Inner Mongolia populations (WNT: 220.09 mg g^–1^ FW; KL: 215.91 mg g^–1^ FW) was significantly higher than that in Xinjiang populations (TKX: 203.02 mg g^–1^ FW; GC: 198.87 mg g^–1^ FW) under SD stress (*p* < 0.001).

The change in soluble protein content of different geographical populations of *S. rostratum* was increased along with drought intensity. In the control, the soluble protein content of the WNT population (21.098 mg g^–1^ FW) was highest, and the GC population (18.63 mg g^–1^ FW) had the lowest content. Under MD stress, Inner Mongolia populations (KL: 28.928 mg g^–1^ FW; WNT: 27.874 mg g^–1^ FW) had a higher soluble protein content than Xinjiang populations (TKX: 26.96 mg g^–1^ FW; GC: 25.918 mg g^–1^ FW), and the soluble protein content of the Xinjiang populations increased the most (*p* < 0.001). Under SD stress, the soluble protein content of the Xinjiang site began to decline compared with that under the MD stress condition, while that in Inner Mongolia populations continued to increase (Figure 5B).

With increased drought stress, the soluble sugar content of different populations of *S. rostratum* increased, reaching a maximum under MD stress treatment (Figure 5C). Under control, LD and MD drought-stress conditions, the soluble sugar content in Inner Mongolia populations was higher than that in Xinjiang populations. Under SD stress, soluble sugar content of the Inner Mongolia populations (WNT: 84.86 mg g^–1^ FW; KL: 81.01 mg g^–1^ FW) decreased slightly, while that in the Xinjiang populations (GC 80.04 mg g^–1^ FW, TKX 80.97 mg g^–1^ FW) continued to increase (*p* < 0.05).

### Relationship Between Responses of *S. rostratum* to Drought Stress by Correlations Analysis and Principal Component Analysis

**FIGURE 6:**
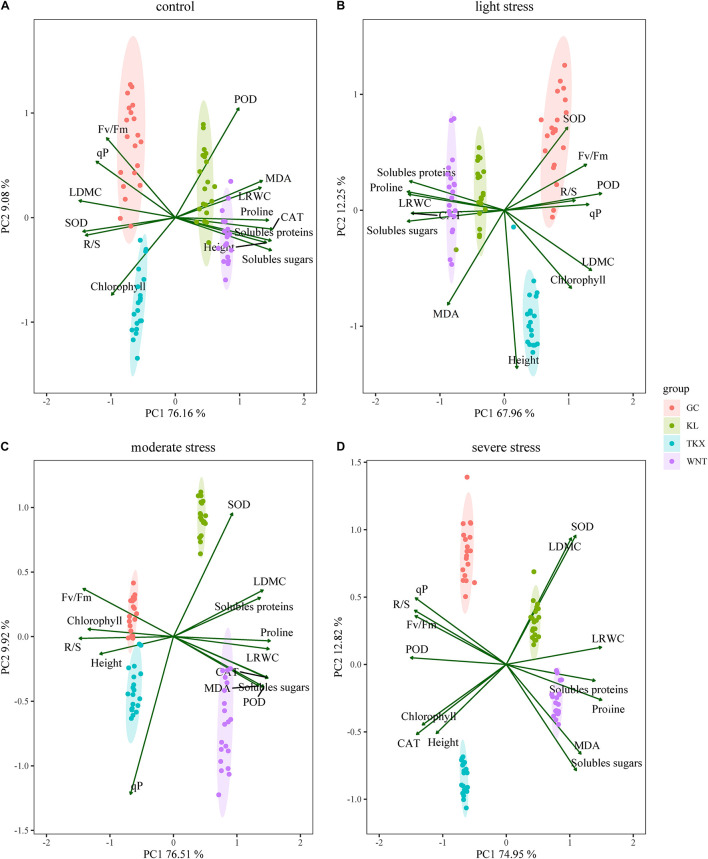
Site score map of two principal components of *S. rostratum*. in different geographical populations under control condition **(A)**, LD stress **(B)**, MD stress **(C)**, and SD stress **(D)** treatments. Principal component analysis variables included plant height (Height), leaf relative water content (LRWC), leaf dry matter content (LDMC), root shoot ratio (R/S), chlorophyll content (Chlorophyll), maximum photochemical efficiency (*F*_v_/*F*_m_), photochemical quenching coefficient (*q*P), malondialdehyde (MDA), superoxide dismutase (SOD), peroxidase (POD), catalase (CAT), proline, soluble protein, and soluble sugar specific activity.

Two principal components were extracted from these measured parameters of each population using the principal component analysis method. In the four PCAs shown, two components with an Eigenvalue equal to or greater than 1 explained a cumulative percentage of variance was over 80.25% (Figure 6). Under CK condition, GC collected site gathered around Fv/Fm and *q*P (Figure 6A), proline was positively correlated with SOD and soluble sugar, but negatively correlated with POD (*p* < 0.01, Supplementary Figure 1A); TKX collected site close to chlorophyll (Figure 6A), MDA was significantly positive correlated with POD and *F*_v_/*F*_m_ (*p* < 0.001); soluble sugar was significantly negative correlated with SOD (*p* < 0.001, Supplementary Figure 1B); KL collected site nearby POD, MDA, CAT and soluble proteins (Figure 6A), *q*P was obviously negative correlated with proline, CAT and height (*p* < 0.001, Supplementary Figure 1C); height was obviously positive correlated with CAT (*p* < 0.001, Supplementary Figure 1C); WNT collected site close to proline, soluble proteins, soluble sugars and height (Figure 6A), correlation between POD and MDA was significant positive (*p* < 0.001, Supplementary Figure 1D).

Under LD stress, the GC nearby SOD, *F*_v_/*F*_m_ and R/S (Figure 6B), soluble sugar was obviously positive correlated with CAT, POD was obviously positive correlated with height (*p* < 0.001, Supplementary Figure 2A); the TKX site close on chlorophyll, LDMC and height (Figure 6B), soluble sugar was significantly negatively correlated with *F*_v_/*F*_m_ (*p* < 0.001, Supplementary Figure 2B); the KL site correlated with soluble proteins, proline and CAT (Figure 6B), the measurement index with significant negative correlation is: proline with MDA and soluble sugar, LDMC with soluble sugar and proline, *F*_v_/*F*_m_ with SOD (*p* < 0.001, Supplementary Figure 2C); the WNT site close to soluble proteins, CAT and soluble sugars (Figure 6B), soluble sugar was negatively correlated with CAT (*p* < 0.01, Supplementary Figure 2D).

Under MD stress, the GC population gathered around *F*_v_/*F*_m_ and R/S (Figure 6C), the soluble proteins is significantly negatively correlated with *q*P, MDA was significantly negatively correlated with LDMC (*p* < 0.001, Supplementary Figure 3A); TKX site close to height (Figure 6C), SOD was negatively correlated with R/S, MDA, and soluble proteins (*p* < 0.01, Supplementary Figure 3B); the KL site nearby SOD, SOD was significantly negatively correlated with R/S (*p* < 0.001, Supplementary Figure 3C); the WNT population gathered around MDA, POD and soluble sugar (Figure 6C), soluble sugar was negatively correlated with CAT (*p* < 0.01, Supplementary Figure 3D).

Under SD stress, the GC site close to *q*P and R/S (Figure 6D), height was significantly negatively correlated with proline, LRWC was significantly negatively correlated with proline and *q*P (*p* < 0.001, Supplementary Figure 4A); TKX site close to height, chlorophyll and CAT (Figure 6D), the measurement index with significant negative correlation is: proline was with SOD and *q*P, chlorophyll with height, soluble proteins with POD (*p* < 0.001, Supplementary Figure 4B). KL site nearby LDMC and SOD (Figure 6D), the measurement index with negative correlation is: POD with *q*P, soluble protein and soluble sugar, MDA with soluble sugar and chlorophyll with CAT (*p* < 0.01, Supplementary Figure 4C), SOD was positively correlated with POD (*p* < 0.01, Supplementary Figure 4C). WNT site nearby proline, MDA and soluble sugars (Figure 6D), proline was significantly negatively correlated with MDA, POD was significantly negatively correlated with *F*_v_/*F*_m_ (*p* < 0.001, Supplementary Figure 4D), and MDA was significantly positively correlated with CAT and soluble proteins (*p* < 0.001, Supplementary Figure 4D). Based upon the principal component loadings, the resistance of Xinjiang population to drought stress is related to chlorophyll fluorescence content and functional traits, however, the resistance of Inner Mongolia population to drought stress is related to the increase of osmotic regulators and LRWC.

## Discussion

The alien invasive plants cushion the selection pressure brought by the new environment through phenotypic plasticity (Feng et al., 2009), adjusting their morphological and physiological characteristics to those compatible with the invasive place (Scheiner, 1993; Pigliucci, 2005). Different populations of invasive plant *Alternanthera philoxeroides* allocated much more biomass to the belowground roots in the terrestrial environment to colonize both aquatic and terrestrial habitats (Geng et al., 2007). At different stages of invasion, invasive plants make local adaptation to the water environment gradient of the invasion site through the changes of a series of functional traits such as physiological response (Rice and Mack, 1991; Leger and Rice, 2007; Liu et al., 2010). In our field investigation, *S. rostratum*, which is distributed in northern and west China, can survive in habitats with different natural environments, especially in the extremely arid areas of Xinjiang, and form dominant communities in a short time, indicating that this invasive plant has strong tolerance to drought stress. Studying its response mechanism to drought stress is the basis for understanding the phenotypic plasticity and adaptive evolution of *S. rostratum* to invasive environment.

### Adaptive Changes in Growth Traits

Plant growth traits are the most direct response to environmental changes. Plants of different geographical populations have different sensitivity to environmental ecological factors. Under stress, invasive plants acquire resources, occupy habitats and enhance invasive energy by changing their morphology, growth, biomass allocation and physiological characteristics (Richards et al., 2006; Hulme, 2007). For instance, when ragweed is subjected to salinity stress, it adapts to drought stress and salt stress environment by changing biomass allocation (performance maintenance) (Onen et al., 2017). The study of 26 different populations of *Chromolaena odorata* showed that this invasive plant adapted to different environments by regulating biomass allocation (Liao et al., 2020). In our research, when the hydrothermal condition was normal (CK condition), the plant height of the Inner Mongolia populations was obviously higher than that of the Xinjiang populations, and the R/S was lower than that of the Xinjiang population, which is consistent with what we have observed in the wild. At sampling sites in Inner Mongolia, *S. rostratum* concentrates more resources on the above-ground part to promote growth, the communities with larger crowns and higher dominance have been formed in the wild, while the underground part is not developed. The plant height of *S. rostratum* at sampling sites in Xinjiang is obviously lower and the crown width is also smaller, but the underground part is significantly longer than those at the sampling sites in Inner Mongolia, which is consistent with the growth characteristics of plants in most arid areas (Silvertown et al., 2015). The LRWC of Inner Mongolia population is higher than that of Xinjiang population in the whole experimental stage, maintaining at the level of 58%, we consider that because the sampling sites in Inner Mongolia are located at the course of seasonal river and edges of desert grassland, the water condition is better than Xinjiang population, LRWC will not change significantly in short-term drought stress. LDMC and R/S were the key traits reflecting plant adaptation to habitat (Milla et al., 2005; van Bodegom et al., 2014). Under drought stress, dry matter will be preferentially distributed to the root system to promote root development and increase the R/S ratio, enhancing drought resistance (Bradbury, 1990). In this study, compared with CK, the R/S of the two populations increased significantly with the increase of stress level, and the R/S of Xinjiang population was higher than that of Inner Mongolia population in the whole experimental process. It indicating that under drought stress, Xinjiang population can give priority to developing roots, improve R/S and obtain more water resources to enhance drought resistance.

### Adaptive Changes in Chlorophyll Fluorescence Parameters

The adaptability of plants to the environment is largely related to their photosynthetic characteristics (Huangfu et al., 2009). The metabolic limitation caused by drought is the primary reason for the decrease of photosynthetic rate. Drought stress can inhibit a series of processes such as electron transfer, light energy conversion, photophosphorylation and dark reaction of photosynthesis (Centritto, 2005; Santos et al., 2017). A research on cereal crops indicated that plants which can retain high chlorophyll content under drought stress condition, could utilize more effective use of light energy to enhance their drought resistance (Sayed, 2003). Photosynthetic Characteristics invasive weed *Flaveria bidentis* has showed the strong photosynthetic ability, its growth period is more suitable for the growth environment of high temperature and drought in summer (Huangfu et al., 2009). In this study, we found that chlorophyll content *of S. rostratum* from two populations in CK were maintained high level and was no significant difference between them. Under LD stress, compares with CK, the chlorophyll content of Inner Mongolia represented obvious downward trend, while the chlorophyll content of Xinjiang population decreased significantly under MD stress, indicating that the chlorophyll content of Inner Mongolia population is more sensitive response to drought stress. Throughout the experiment, the chlorophyll content of TKX sampling point has always been maintained at a high level, and under SD stress, the chlorophyll content of TKX sampling point is significantly higher than that of other populations. We believe that the main reason is that the *S. rostratum* of TKX sampling point grows in roadside habitats in extreme arid areas, and the drought pressure in its original environment is greater than that of other populations. During the homogeneous garden experiment, maintaining a high chlorophyll content is the response measure of TKX sampling point to resist drought stress. Furthermore, it shows strong drought resistance of TKX sampling point.

Chlorophyll fluorescence are helpful to reveal the relationship between plant photosynthetic physiology and drought stress (van Kooten and Snel, 1990). Chlorophyll fluorescence parameters can quickly, sensitively and non-invasively reflect the state of PS II, they are important parameters for analyzing and evaluating the function of plant photosynthetic mechanism and the impact of environmental stress (Banks, 2018; Zhang et al., 2020). In our study, the *F*_v_/*F*_m_ values of different populations of *S. rostratum* had no significant change compared with the control under LD stress. Under MD stress, the *F*_v_/*F*_m_ and *q*P values of Inner Mongolia population and Xinjiang population showed an obvious downward trend, indicating that the PS II reaction center of different populations of *S. rostratum* was destroyed and the photochemical activity of PS II was inhibited under moderate drought stress, it reduces the primary light energy conversion efficiency of PS II, impairs the potential activity of PS II, and limits the normal progress of photosynthesis. This result is consistent with the photosynthetic characteristics of other plants distributed in arid and semi-arid areas of China under drought stress (Chen et al., 2019; Wang et al., 2019).

### Adaptive Changes in MDA and Antioxidant Enzyme Activity

Under severe temperature, salt, radiation or drought stress, the metabolic balance between production and elimination of reactive oxygen species in plants is broken (Chen et al., 2019). The resulting ROS accumulation leads to membrane lipid peroxidation, and malondialdehyde is the product of membrane lipid peroxidation. The resulting ROS accumulation leads to membrane lipid peroxidation (Chen et al., 2011), and malondialdehyde (MDA) is its product. The content of MDA indicates the degree of stress injury of plant cells (Draper and Hadley, 1990), which is considered to be an excellent marker of oxidative stress (Del Rio et al., 2005). The study on the physiological and ecological characteristics of invasive plants under various stress conditions showed that under drought stress (Dong et al., 2014), waterlogged condition (Wang et al., 2017), saline alkali stress (Wang et al., 2019) and heavy metal stress (Zhang et al., 2020), the MDA content of alien invasive plants could increase rapidly, and maintained a high level of membrane lipid peroxidation at the maximum stress.

Under CK conditions, there was no significant difference in MDA content among various populations. Under MD stress, the MDA content of Inner Mongolia population began to be significantly higher than that of Xinjiang population, indicating that MD stress led to a large accumulation of ROS in cells of *S. rostratum*, which exacerbated membrane lipid peroxidation. At the same time, the leaves of Inner Mongolia population were significantly damaged by ROS. Under SD stress, the MDA content of various groups of *S. rostratum* increased significantly compared with CK, indicating that under SD stress, a large amount of MDA accumulated and caused more and more damage to the cell membrane. Among them, the MDA content of WNT sampling point is significantly higher than that of other populations, indicating that under SD conditions, the MDA accumulation rate of Inner Mongolia population is higher than Xinjiang population, and it is more sensitive to drought stress.

Under stress, ROS are produced, such as hydrogen peroxide (H_2_O_2_), hydroxyl radical (OH^–^), singlet oxygen (O^1–^), and superoxide radical (O^2–^) (Mittler, 2002). To avoid or alleviate cell damage caused by ROS, plants stimulate their antioxidant enzyme system, and these protective enzymes are closely related to plant stress resistance. SOD activity is the first defense against membrane lipid peroxidation induced by ROS, which catalyzes O^2–^ dis-mutation and conversion into H_2_O_2_ and O_2_. CAT and POD activities are mainly responsible for removing this H_2_O_2_, thereby further lowering ROS levels (Karataş et al., 2012). In our study, the SOD activity of various groups of *S. rostratum* increased with the increase of stress gradient, reached the maximum under SD stress, and the SOD activity of Inner Mongolia population was greater than that of Xinjiang population, indicating that SOD activity played an important role in the whole drought stress stage and enhanced the drought resistance of *S. rostratum*. However, the changes of POD and CAT activities were significantly different between Xinjiang and Inner Mongolia populations under SD stress. Compared with CK, the activities of POD and CAT in Xinjiang population increased significantly under SD stress, indicating that these two enzymes play an important protective role in protecting Xinjiang population from SD stress. On the contrary, the activities of SOD and CAT in Inner Mongolia population were significantly lower than that in Xinjiang population under SD stress, illustrating that the protective effect of SOD and CAT activities on Inner Mongolia population was weakened, that shows the two different geographical populations of *S. rostratum* took different stress resistance mechanisms under SD stress.

### Adaptive Changes of Osmoregulatory Substances

Osmotic regulation by accumulating organic solutes is an important mechanism for plants to respond to drought stress (Morgan and Condon, 1986). Plants will produce osmoregulation substances after water stress, cells hold turgor by accumulating large amounts of osmoregulation substances, maintaining plant physiological processes (Flexas and Medrano, 2002; Lawson et al., 2003), as to improve the drought resistance of plants. Proline is an important osmotic regulator in plants. Proline accumulation is the first response of plants to water stress, which support subcellular structure, scavenge free radicals, and regulate cell redox potential under water stress (Szabados and Savouré, 2010). Proline accumulation is a general response to stress, many plant species have increased proline levels under drought stress (Muscolo et al., 2014; Azmat and Moin, 2019).

We found that the proline content of the two populations increased with the increase of stress gradient. The proline content of Inner Mongolia population was greater than that of Xinjiang population under all drought stress conditions, indicating that proline plays an important role in the drought defense mechanism of *S. rostratum*. The soluble protein content of Xinjiang population increased with the increase of drought stress gradient, and reached the maximum under MD stress, but showed a downward trend under SD stress, indicating that for Xinjiang population, soluble protein played a positive role under MD stress, but its effectiveness decreased under severe stress. On the contrary, the soluble protein content of Inner Mongolia population reached the maximum under SD stress, which exceeded that of Xinjiang population, indicating that the increase of soluble protein is a drought resistance regulation mechanism of Inner Mongolia population. With the increase of drought stress, the soluble sugar content of various groups of *S. rostratum* increased, illustrating that these sugars play a role in drought stress. Under SD stress, the soluble sugar content of KL sampling point did not increase, indicated that *S. rostratum* at KL sampling point could not continue to produce enough soluble sugar to resist drought stress. It is worth mentioning that the accumulation of soluble protein under stress promotes osmotic regulation, which is a storage form of plant nitrogen (An et al., 2011), different populations of *S. rostratum* have different feedback of soluble proteins under drought stress. Is this the embodiment of resource capture ability and utilization efficiency of *S. rostratum* as an invasive plant (Feng et al., 2009)? Does *S. rostratum* in different habitats adopt different nitrogen distribution mechanisms to drought stress (Feng et al., 2009)? We need further study.

## Conclusion

In order to explain the invasion mechanism and its ecological process more objectively and accurately, it is necessary to comprehensively analyze the individual ecological characteristics of plants and their interaction with environmental factors in the invasion areas. Based on the above analyses, it is concluded that the physiological responses of *S. rostratum* invading different habitats in northern China to drought stress were significantly different. The Xinjiang population was more drought-resistance than that of the Inner Mongolia population. *S. rostratum* can be widely adapted to both harsh and suitable habitats through phenotypic plasticity.

However, with the increasing impact of global climate change and human activities, the potential long-term effects of environmental factors on invasive mechanisms and adaptive evolution of invasive plants are still needed to reveal the genetic, physiological and growth plasticity of invaded plants to the changing factors, naturally and/or anthropogenically.

## Data Availability Statement

The original contributions presented in the study are included in the article/Supplementary Material, further inquiries can be directed to the corresponding author/s.

## Author Contributions

HY and XZ conceived the research plan. HY, XZ, and WH conceptualized and planned the experiments. HY conducted the experiments. HY, JZ, and YH performed data analysis and wrote the first draft of the manuscript. HY, XZ, WH, JZ, and YH contributed to manuscript editing, revision, reading, and approval of the submitted version. All authors contributed to the article and approved the submitted version.

## Conflict of Interest

The authors declare that the research was conducted in the absence of any commercial or financial relationships that could be construed as a potential conflict of interest.

## Publisher’s Note

All claims expressed in this article are solely those of the authors and do not necessarily represent those of their affiliated organizations, or those of the publisher, the editors and the reviewers. Any product that may be evaluated in this article, or claim that may be made by its manufacturer, is not guaranteed or endorsed by the publisher.

## Supplementary Material

Supplementary Figure 1 Pearson’s correlation coefficient (*r*) for the relationships among the physiological characteristics for different geographical populations of *S. rostratum* under the control condition. **(A)** GC site; **(B)** TKX site; **(C)** KL site; and **(D)** WNT site. ^∗∗∗^ and ^∗∗^ indicate significant differences at *p* < 0.001 and *p* < 0.01 levels, respectively.

Supplementary Figure 2 Pearson’s correlation coefficient (*r*) for the relationships among the physiological characteristics for different geographical populations of *S. rostratum* under light drought stress (LD) conditions. **(A)** GC collected site; **(B)** TKX site; **(C)** KL site; and **(D)** WNT site. ^∗∗∗^ and ^∗∗^ indicate significant differences at *p* < 0.001 and *p* < 0.01 levels, respectively.

Supplementary Figure 3 Pearson’s correlation coefficient (*r*) for the relationships among the physiological characteristics for different geographical populations of *S. rostratum* under moderate drought stress (MD) condition. **(A)** GC site; **(B)** TKX site; **(C)** KL site; and **(D)** WNT site. ^∗∗∗^ and ^∗∗^ indicate significant differences at *p* < 0.001 and *p* < 0.01 levels, respectively.

Supplementary Figure 4 Pearson’s correlation coefficient (*r*) for the relationships among the physiological characteristics for different geographical populations of *S. rostratum* under severe drought stress (SD) condition. **(A)** GC collected site; **(B)** TKX collected site; **(C)** KL collected site; and **(D)** WNT collected site. ^∗∗∗^ and ^∗∗^ indicate significant differences at *p* < 0.001 and *p* < 0.01 levels, respectively.

Supplementary Table 1 List of non-standard abbreviations.

Supplementary Table 2 List of related trait features and calculation formula.

Supplementary Table 3 ANOVA outputs for individual and interactive effects of population and drought-stress treatment on plant functional traits, chlorophyll fluorescence, antioxidant enzyme activity, and osmoregulatory substances.
