# Supplementary figures and images for: Drought Stress Influences the Growth and Physiological Characteristics of Solanum rostratum Dunal Seedlings From Different Geographical Populations in China
Source: Front Plant Sci. 2021 Nov 16;12:733268. doi: 10.3389/fpls.2021.733268 (PMC8637895; doi:10.3389/fpls.2021.733268)

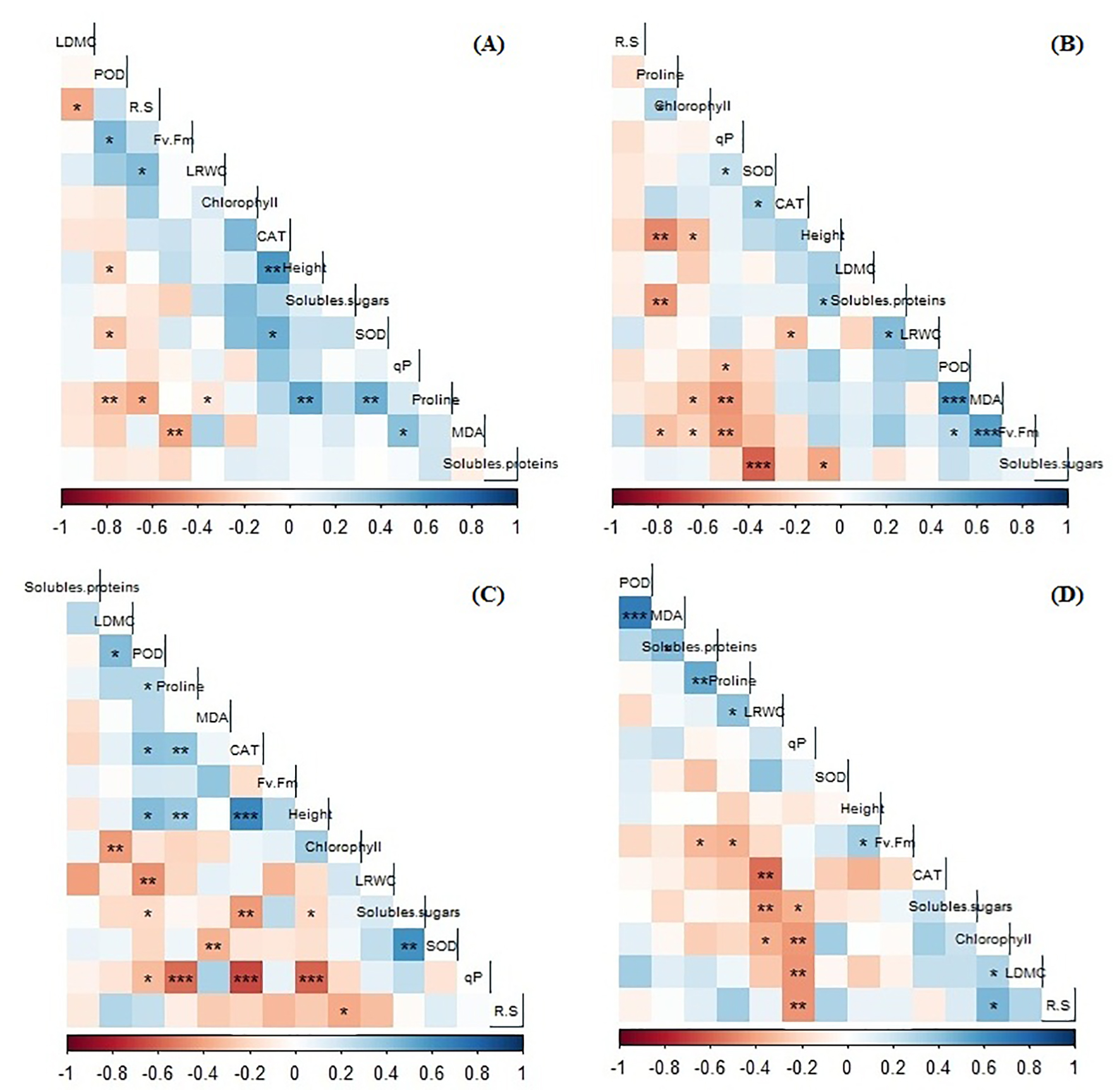

Supplement: Supplementary Figure 1 — Pearson’s correlation coefficient (r) for the relationships among the physiological characteristics for different geographical populations of S. rostratum under the control condition. (A) GC site; (B) TKX site; (C) KL site; and (D) WNT site. ∗∗∗ and ∗∗ indicate significant differences at p < 0.001 and p < 0.01 levels, respectively. [file Image_1.JPEG]

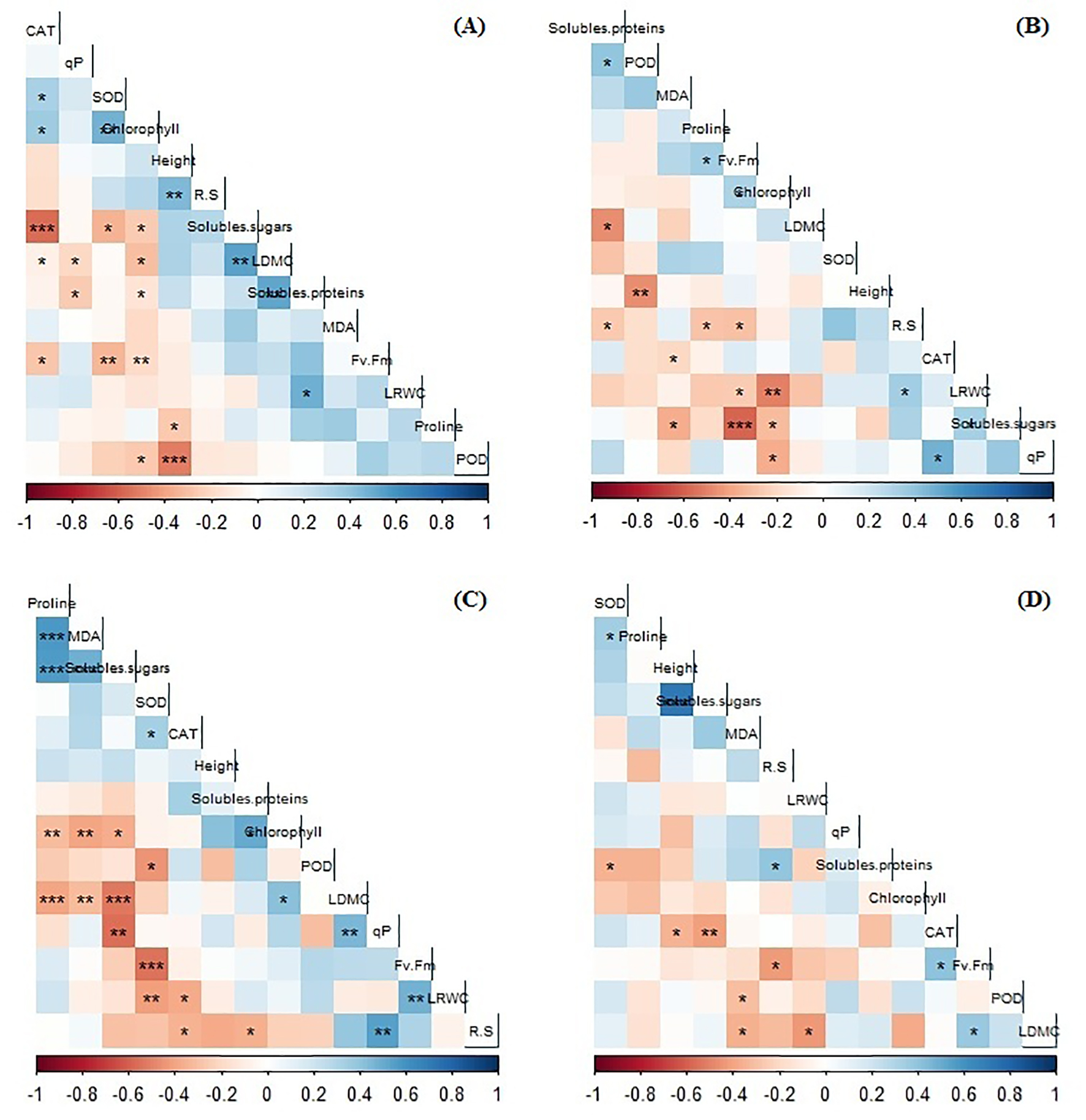

Supplement: Supplementary Figure 2 — Pearson’s correlation coefficient (r) for the relationships among the physiological characteristics for different geographical populations of S. rostratum under light drought stress (LD) conditions. (A) GC collected site; (B) TKX site; (C) KL site; and (D) WNT site. ∗∗∗ and ∗∗ indicate significant differences at p < 0.001 and p < 0.01 levels, respectively. [file Image_2.JPEG]

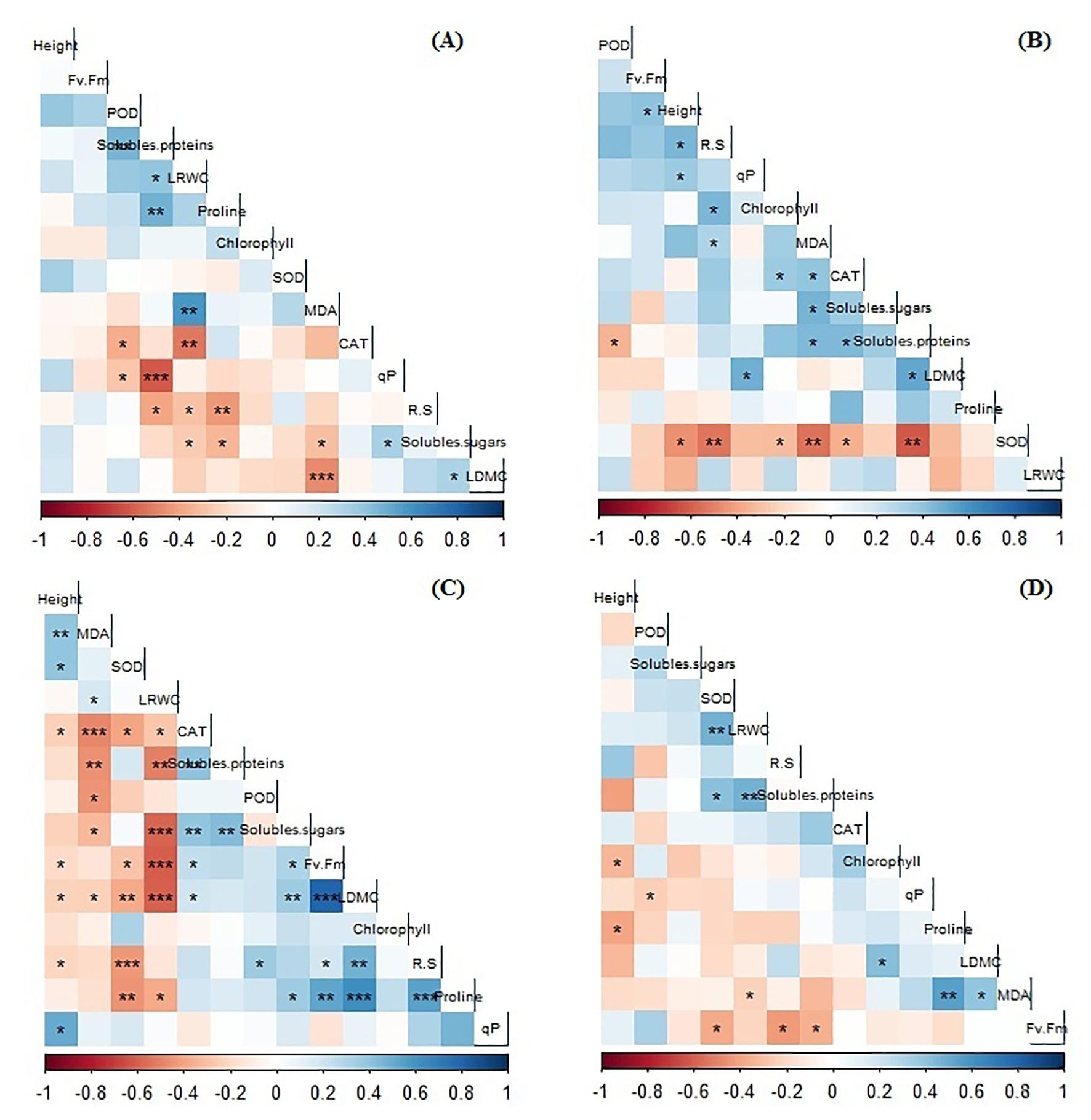

Supplement: Supplementary Figure 3 — Pearson’s correlation coefficient (r) for the relationships among the physiological characteristics for different geographical populations of S. rostratum under moderate drought stress (MD) condition. (A) GC site; (B) TKX site; (C) KL site; and (D) WNT site. ∗∗∗ and ∗∗ indicate significant differences at p < 0.001 and p < 0.01 levels, respectively. [file Image_3.JPEG]

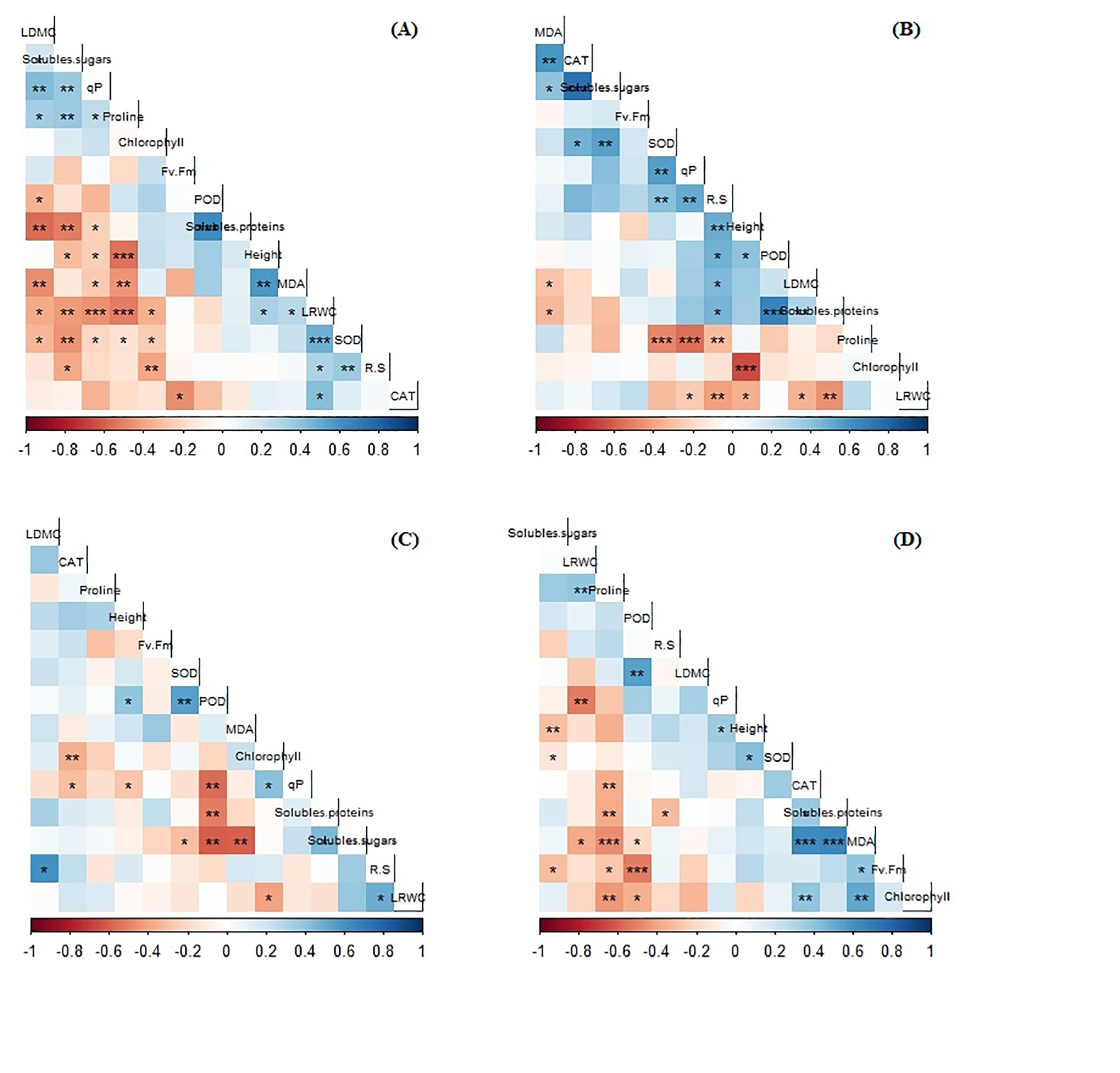

Supplement: Supplementary Figure 4 — Pearson’s correlation coefficient (r) for the relationships among the physiological characteristics for different geographical populations of S. rostratum under severe drought stress (SD) condition. (A) GC collected site; (B) TKX collected site; (C) KL collected site; and (D) WNT collected site. ∗∗∗ and ∗∗ indicate significant differences at p < 0.001 and p < 0.01 levels, respectively. [file Image_4.JPEG]
