# Supplementary material for: Drought Stress Influences the Growth and Physiological Characteristics of Solanum rostratum Dunal Seedlings From Different Geographical Populations in China
Source: Front Plant Sci. 2021 Nov 16;12:733268. doi: 10.3389/fpls.2021.733268 (PMC8637895; doi:10.3389/fpls.2021.733268)
Supplement: Supplementary Table 1 — List of non-standard abbreviations. [file Table_1.docx]

**Supplementary Table 1.** List of non-standard abbreviations.

| **Abbreviation** | **Full form** |
| --- | --- |
| *A*_450_ | absorbance of enzyme solution at 470 nm |
| *A*_532_ | absorbance of enzyme solution at 532 nm |
| *A*_600_ | absorbance of enzyme solution at 600 nm |
| AB | aboveground biomass |
| APX | ascorbate peroxidase |
| CAT | catalase |
| CK | control |
| DHAR | dehydroalbumin reductase |
| DW | dry weight of the leaf |
| *F*_m_ | maximum fluorescence |
| *F*_m_′ | maximum fluorescence under light adaptation |
| *F*_o_ | initial fluorescence |
| *F*_s_ | stable fluorescence |
| *F*_v_ | variable fluorescence |
| FW | fresh weight of the leaf |
| GC | Gaochang district |
| H_2_O_2_ | hydrogen peroxide |
| KL | Kailu County |
| LD | light drought |
| LDMC | leaf dry matter content |
| LRWC | leaf relative water content |
| MD | moderate drought |
| MDA | malondialdehyde |
| NBT | nitro blue tetrazole |
| NIEER | Northwest Institute of Eco-Environment and Resources |
| O^1−^ | singlet oxygen |
| O^2−^ | superoxide radical |
| OH^−^ | hydroxyl radical |
| PCA | principal component analysis |
| PEG | polyethylene glycol |
| POD | peroxidase |
| *q*P | quenching coefficient |
| R/S | root/shoot ratio |
| ROS | reactive oxygen species |
| SD | severe drought |
| SFW | saturated fresh weight of leaf |
| SOD | superoxide dismutase |
| SP | soluble protein |
| SS | soluble sugars |
| TBA | thiobarbituric acid |
| TCA | trichloroacetic acid |
| TKX | Tuokexun County |
| UB | underground biomass |
| V_extract_ | extract volumn |
| WNT | Ongniud Banner |
