# Supplementary material for: Drought Stress Influences the Growth and Physiological Characteristics of Solanum rostratum Dunal Seedlings From Different Geographical Populations in China
Source: Front Plant Sci. 2021 Nov 16;12:733268. doi: 10.3389/fpls.2021.733268 (PMC8637895; doi:10.3389/fpls.2021.733268)
Supplement: Supplementary Table 2 — List of related trait features and calculation formula. [file Table_2.DOCX]

**Supplementary Table 2.** List of related trait features and calculation formula.

| **Trait (Abbreviation)** | **Equation** | **Unit** |
| --- | --- | --- |
| Leaf relative water content (LRWC) | LRWC = (FW – DW) / (SFW – DW) | % |
| Leaf dry matter content (LDMC) | LDMC = DW / SFW | mg/g |
| Root/shoot ratio | R/S = UB / AB | % |
| Variable fluorescence (*F*_v_) | *F*_v_ = *F*_m_ – *F*_o_ |  |
| Maximum photochemical efficiency | (*F*_v_ / *F*_m_) = (*F*_m_ – *F*_o_) / *F*_m_ |  |
| Photochemical quenching coefficient | *q*P = (*F*_m_′–*F*_t_) / (*F*_m_ – *F*_o_) |  |
| Malondialdehyde concentration (CONC_MDA_) | CONC_MDA_ = 6.45 × (*A*_532_ – *A*_600_) – 0.56 *A*_450_ | mol / L |
| Malondialdehyde content (CONT_MDA_) | CONT_MDA_ = (CONC_MDA_ ×V_extract_) / (FW × 1000) | mmol/g FW |
