# Supplementary material for: Drought Stress Influences the Growth and Physiological Characteristics of Solanum rostratum Dunal Seedlings From Different Geographical Populations in China
Source: Front Plant Sci. 2021 Nov 16;12:733268. doi: 10.3389/fpls.2021.733268 (PMC8637895; doi:10.3389/fpls.2021.733268)
Supplement: Supplementary Table 3 — ANOVA outputs for individual and interactive effects of population and drought-stress treatment on plant functional traits, chlorophyll fluorescence, antioxidant enzyme activity, and osmoregulatory substances. [file Table_3.DOCX]

**Supplementary Table 3.** The effects of population, drought-stress treatment, and their interaction on plant functional traits, chlorophyll fluorescence, antioxidant enzyme activity, and osmoregulatory substances of *S. rostratum* from different geographical populations (two-way ANOVAs).

| **Index** | **Concentration** |  | **Location** |  | **Location×concentration** |  |
| --- | --- | --- | --- | --- | --- | --- |
|  | *F* | *P* | *F* | *P* | *F* | *P* |
| Height | 731.675 | 0.001 | 2.316 | 0.084 | 5.070 | 0.001 |
| LRWC | 15265.393 | 0.001 | 516.672 | 0.001 | 19.668 | 0.001 |
| LDMC | 4065.242 | 0.001 | 119.356 | 0.001 | 507.705 | 0.001 |
| R/S | 7900.491 | 0.001 | 1101.250 | 0.001 | 21.364 | 0.001 |
| Chlorophyll | 900.245 | 0.001 | 116.834 | 0.001 | 7.593 | 0.001 |
| *F*_v_/*F*_m_ | 1230.365 | 0.001 | 87.619 | 0.001 | 2.685 | 0.010 |
| *q*P | 475.007 | 0.001 | 90.280 | 0.001 | 9.479 | 0.001 |
| MDA | 4712.220 | 0.001 | 59.400 | 0.001 | 8.796 | 0.001 |
| SOD | 563664.095 | 0.001 | 813.064 | 0.001 | 914.337 | 0.001 |
| POD | 75724.152 | 0.001 | 486.754 | 0.001 | 739.307 | 0.001 |
| CAT | 189008.937 | 0.001 | 673.505 | 0.001 | 450.071 | 0.001 |
| Proline | 458188.179 | 0.001 | 3942.110 | 0.001 | 330.218 | 0.001 |
| Soluble proteins | 2475.698 | 0.001 | 373.144 | 0.001 | 35.830 | 0.001 |
| Soluble sugars | 28435.753 | 0.001 | 1494.964 | 0.001 | 44.239 | 0.001 |
